# Association between the neutrophil percentage-to-albumin ratio and pelvic adhesion severity in endometriosis: A retrospective cross-sectional study

**DOI:** 10.1371/journal.pone.0337077

**Published:** 2025-12-02

**Authors:** Xiurong Tan, Hong Zhang, Chun Yang, Junhui Chen

**Affiliations:** 1 Department of Obstetrics and Gynecology, Sichuan Science City Hospital, China; 2 Department of Obstetrics and Gynecology, The First Affiliated Hospital of SuZhou University, China; Athens Medical Group, Psychiko Clinic, GREECE

## Abstract

**Study background:**

Preoperative assessment of the severity of pelvic adhesions in endometriosis remains challenging. Inflammation may drive adhesion formation, and the predictive value of the neutrophil percentage-to-albumin ratio (NPAR, the ratio multiplied by 10 for analysis), an emerging biomarker of systemic inflammation, in endometriosis pelvic adhesions has not been evaluated.

**Methods:**

In this study, we retrospectively analyzed the data of 246 patients with histologically confirmed ovarian endometriotic cysts and statistically analyzed NPAR. We evaluated the correlation between NPAR and pelvic adhesion severity in patients with endometriosis using logistic regression models and logistic fitted curves (to evaluate the dose-response relationships). Additionally, the predictive efficacy of NPAR for pelvic adhesion severity was assessed using a receiver operating characteristic (ROC) curve analysis.

**Results:**

NPAR was positively correlated with the severity of pelvic adhesions, demonstrating statistically significant associations with moderate-to-severe pelvic adhesions in all models when utilized as a continuous variable, and the risk of moderate-to-severe pelvic adhesions increased by 82% for every 10-unit increase in NPAR (Odds Ratio, OR = 1.82); when used as a categorical variable, the risk of moderate-to-severe pelvic adhesions was remarkably increased in the highest tertile of NPAR. ROC curve analysis demonstrated that the area under the curve of the NPAR score was 0.717, which was superior to that of the CA125 (Area Under the Curve,AUC = 0.564).

**Conclusion:**

NPAR is an independent predictor of moderate to severe pelvic adhesions in patients with endometriosis and is superior to that of CA125. As an easily accessible blood-based biomarker, NPAR may be useful for preoperative risk stratification, guiding surgical planning and individualized treatment decisions.

## Introduction

Endometriosis is a common gynecological condition characterized by the presence of endometrial tissue (glands and stroma) outside the uterine cavity [1]. Its prevalence is estimated to be 2–10% in the general female population and up to 50% in infertile women [2–4]. Pelvic adhesions are a common clinical manifestation of endometriosis [5] and are one of the most important causes of infertility or ectopic pregnancy in women [6]. Severe pelvic adhesions not only increase the difficulty and duration of surgery but may also be a limiting factor for laparoscopic surgery or even lead to intermediate open surgery [7]. Therefore, accurate preoperative assessment of adhesion severity is vital for optimizing treatment strategies, selecting appropriate surgical approaches, and improving patient prognoses.

Transvaginal ultrasonography is a widely employed clinical tool for assessing pelvic adhesions in patients with endometriosis [8,9]. However, growing evidence suggests that the size of endometriotic lesions does not significantly correlate with the severity of adhesions, indicating that imaging alone may not be sufficient for a comprehensive assessment. Additionally, imaging techniques have inherent limitations such as the inability to detect microscopic adhesions and reliance on operator expertise. Therefore, the identification of a more reliable and validated evaluation method is essential to enhance the clinical management of endometriosis.

Recent advances in the understanding of the pathophysiology of endometriosis have emphasized the role of the inflammatory response in adhesion formation. Inflammatory biomarkers, such as the neutrophil percentage-to-albumin ratio (NPAR), have emerged as promising tools for assessing systemic inflammation and infection [10]. However, the relationship between NPAR and pelvic adhesions in endometriosis remains unclear. This study aimed to investigate the predictive value of NPAR in assessing the severity of adhesions in patients with endometriosis to provide a more accurate and accessible clinical assessment tool.

## Materials and methods

### Ethical approval

Retrospective data collection and analysis were approved by the Ethics Committee of the First Affiliated Hospital of Soochow University (Approval No.: 2024 Ethical Review Approval No. 723). The requirement for informed consent was waived due to the retrospective nature of the study.

### Study population

We enrolled 246 patients who underwent laparoscopic surgery and were pathologically diagnosed with ovarian endometriosis at the Department of Gynecology, First Affiliated Hospital of Soochow University, between 01 May 2022 and 30 April 2024. The data for this research were accessed for analysis between 01/06/2024 and 30/06/2024. Authors had access to potentially identifying information during the data collection phase for the purpose of scoring adhesion severity from surgical records. All data were de-identified immediately after collection and prior to statistical analysis, and the authors had no access to information that could identify individual participants at any stage of the study. Patients were excluded if they satisfied any of the following criteria: age < 18 years or postmenopausal status, adnexal mass torsion or rupture, clinical suspicion or histopathological diagnosis of uterine fibroids/adenomyosis, history of pelvic inflammatory disease, previous abdominopelvic surgery, acute/chronic inflammatory diseases, abnormal liver function, hematologic disorders, immune system diseases, any type of cancer, hypertension, diabetes mellitus, or receipt of hormonal therapy within the past 6 months.

### Data collection

The laboratory parameters collected included lymphocyte count, eosinophil count, basophil count, monocyte count, neutrophil count, neutrophil percentage, platelet count, fibrinogen level, high-sensitivity C-reactive protein, albumin level, and CA125 level. The methodologies for these measurements are detailed in the ‘Laboratory Methods’ section. Derived biomarkers were calculated as follows: The neutrophil-to-lymphocyte ratio (NLR) was calculated as the absolute neutrophil count divided by the absolute lymphocyte count. The neutrophil percentage-to-albumin ratio (NPAR) was defined as the neutrophil percentage divided by the serum albumin level (g/L) and multiplied by 10 for analysis. All peripheral blood samples were collected during the preoperative admission period, within 7 days before the scheduled laparoscopic surgery, and the menstrual phase was avoided to minimize the potential physiological fluctuations in inflammatory markers and CA125 levels.

Clinical data extracted from the electronic medical records included age, gravidity, parity, BMI (calculated as weight in kilograms divided by height in meters squared), and cyst size.

Pelvic adhesion status was assessed from surgical records in the electronic medical records. We quantified adhesion severity using a previously established scoring system [11,12] that evaluated the extent and density of adhesions, pouch of Douglas (POD) closure, adhesions between the adnexa and surrounding tissues, and tubal fimbrial occlusion. Specific quantifications were as follows: (1) extent of adhesions: score 1, adhesions <25% of the surface area; score 2, adhesions 26%–50% of the surface area; and score 3, adhesions >50% of the surface area. (2) Degree of adhesion: 1 point, loose adhesions and/or no vascular adhesions; 2 points, vascular adhesions and/or dense vascular adhesions; 3 points, dense adhesions and/or adhesions without tissue gaps. (3) POD closure: 0 point, no closure; 1 point, partial closure; 2 points, complete closure. (4) Ovarian adhesions: 0 points, no adhesions; 1 point, unilateral adhesions; 2 points, bilateral adhesions. (5) Tubal adhesion: 0 points, no adhesion; 1 point, unilateral adhesion; 2 points, bilateral adhesion. (6) Tubal atresia: 0 points, no atresia; 1 point, unilateral atresia; 2 points, bilateral atresia. The scores of each index were summed to form the pelvic adhesion score, and the severity of adhesion was quantified as follows: 0–1, no adhesion; 2–5, mild adhesion; 6–9, moderate adhesion; 10–14, severe adhesion.

### Laboratory methods

Peripheral blood samples were collected preoperatively during a non-menstrual phase. A complete blood count with differential (providing neutrophil percentage and absolute counts of neutrophils, lymphocytes, monocytes, eosinophils, basophils, and platelets) and high-sensitivity C-reactive protein (hs-CRP) were analyzed on a Mindray BC-5800 automated hematology analyzer. Plasma fibrinogen was measured via the Clauss method on a Sysmex CS-5100 coagulation analyzer. Serum albumin and CA125 were quantified using the bromocresol green method and electrochemiluminescence immunoassay, respectively.

### Statistical analysis methods

A retrospective cross-sectional study design was used, and data collection and analysis methods followed the norms of observational studies. According to the data type and distribution characteristics, for continuous variable data, normally distributed variables were presented as mean ± standard deviation, while skewed distribution variables were presented as median (interquartile range); categorical variables were presented as frequency or percentage. For baseline comparisons, t-tests or one-way analysis of variance were used to test for statistical differences between groups for continuous variables. For categorical variables, chi-square tests or Fisher’s exact tests were employed to evaluate differences between groups.

We calculated odds ratios (ORs) and their 95% confidence intervals (CIs) using logistic regression analysis to evaluate the correlation between NPAR and pelvic adhesions in patients with endometriosis using four stepwise adjusted models: model 1, unadjusted; model 2, adjusted for age, body mass index, gestational age, and number of deliveries; model 3 was further adjusted for lymphocyte count, neutrophil count, platelet count, and fibrinogen;and model 4, based on model 3, which further included CA125 and NLR as adjusted variables to more fully control for the effects of potential confounders. The selection of covariates for multivariable logistic regression models was based on a combination of clinical relevance from the existing literature and statistical considerations. Variables known or suspected to be associated with either inflammation or adhesion formation were considered. Confounding variables were screened based on (i) significance (*P* < 0.1) in one-way analysis, (ii) >10% change in the odds ratio after inclusion in the model, and (iii) clinical significance reported in previous literature. Logistic fitting curves were utilized to assess the association between NPAR and pelvic adhesions in patients with endometriosis.

Additionally, we examined the effects of age (<40 years or ≥40 years), body mass index (BMI) (<24 kg/m^2^ or ≥24 kg/m^2^), and cyst size (<6 cm or ≥6 cm) using stratified and interaction analyses to assess the robustness of the findings. Likelihood ratio tests were used to assess the relationship between NPAR and stratified variables. The screening of the subgroups for confounding variables remained consistent with those screened in Model 4.

To further evaluate the predictive value of NPAR for moderate-to-severe pelvic adhesions, we conducted receiver operating characteristic (ROC) curve analysis. The area under the curve (AUC) was calculated to assess the predictive performance for moderate-to-severe pelvic adhesions in patients with endometriosis. An AUC value > 0.7 indicates that the marker has diagnostic value, and diagnostic accuracy increases as the AUC approaches 1.

All statistical analyses were performed using the Free Statistics Analysis Platform (version 1.9.2; Beijing, China; http://www.clinicalscientists.cn/freestatistics). Free Statistics is a software package that provides a user-friendly interface for common analysis and data visualization. It uses R as the underlying statistical engine and a graphical user interface (GUI) developed in Python, allowing for easily reproducible analyses and interactive calculations. For the purpose of this study, statistical significance was set at a p-value of < 0.05.

## Results

### Subject characteristics

A total of 246 patients with endometriosis were included in this study. Based on the pelvic adhesion score, patients were categorized into a no-to-mild adhesion group (score 0–5) and a moderate-to-severe adhesion group (score 6–14), with the severity of pelvic adhesions serving as the outcome index. Patients were categorized into three groups based on the NPAR tertiles (Table 1). Table 1 summarizes the baseline characteristics of the participants. The mean levels of NPAR in each group were 12.4 ± 0.9, 14.3 ± 0.5, and 16.8 ± 1.4. Patients with high levels of NPAR were the oldest, and as the NPAR values increased, the patients demonstrated elevated levels of ultrasensitive C-reactive protein, eosinophil count, neutrophil count, fibrinogen, CA125, and NLR, whereas lymphocyte counts and albumin decreased.

**Table 1:** Characteristics of study patients by neutrophil percentage-to-albumin ratio (NPAR) tertiles.

Covariates	Total (n = 246)	NPAR Tertiles			*P* Value
		T1 (n = 82)	T2 (n = 82)	T3 (n = 82)	
Age, years	36.7 ± 6.2	35.5 ± 6.2	36.9 ± 5.8	37.9 ± 6.3	**0.04**
BMI, kg/m^2^	22.0 (20.0, 24.5)	21.9(19.6, 24.1)	22.0 (19.9, 24.1)	22.7 (20.4, 25.6)	0.165
Gravidity	2.0 (1.0, 3.0)	1.5 (0.0, 2.0)	2.0 (1.0, 2.0)	2.0 (1.0, 3.0)	0.285
Parity	1.0 (1.0, 1.0)	1.0 (0.0, 1.0)	1.0 (1.0, 1.0)	1.0 (1.0, 1.0)	0.787
hs-CRP,mg/L	1.0 (0.6, 2.3)	0.7 (0.6, 1.3)	1.1 (0.6, 2.2)	1.3 (0.6, 4.0)	**0.001**
EO,10^9^/L	0.1 (0.0, 0.1)	0.1 (0.1, 0.2)	0.1 (0.0, 0.1)	0.1 (0.0, 0.1)	**< 0.001**
LYMPH,10^9^/L	1.6 (1.3, 2.0)	1.9 (1.6, 2.3)	1.6 (1.3, 1.9)	1.4 (1.2, 1.6)	**< 0.001**
MONO,10^9^/L	0.3 ± 0.1	0.3 ± 0.1	0.3 ± 0.1	0.4 ± 0.1	0.286
NEUT,10^9^/L	3.8 ± 1.7	2.9 ± 0.9	3.6 ± 1.5	4.8 ± 2.0	**< 0.001**
PLT, 10^9/^L	251.9 ± 65.4	243.7 ± 52.1	256.9 ± 62.6	255.2 ± 78.7	0.373
FIB,g/L	2.8 (2.5, 3.2)	2.7 (2.5, 3.0)	2.8 (2.6, 3.1)	3.0 (2.7, 3.6)	**< 0.001**
NLR	2.4 ± 1.3	1.6 ± 0.5	2.2 ± 0.8	3.4 ± 1.5	**< 0.001**
NPAR	14.5 ± 2.1	12.4 ± 0.9	14.3 ± 0.5	16.8 ± 1.4	**< 0.001**
Albumin,g/L	43.2 ± 3.5	44.1 ± 3.5	43.2 ± 3.6	42.3 ± 3.3	**0.003**
CA125,U/mL	58.2(35.4, 105.7)	49.0(28.0, 76.0)	60.4(35.9, 108.0)	60.4(42.1, 129.3)	**0.007**
CA199,U/mL	35.6 (17.1, 86.6)	34.6(16.1, 87.6)	34.5 (12.6, 77.3)	43.1(20.1, 101.9)	0.726
Cyst size,cm	7.0 (6.0, 9.0)	7.0 (6.0, 8.0)	8.0 (6.0, 9.0)	7.0 (6.0, 9.0)	0.146

TI-T3:NPAR Tertiles;BMI, body mass index;hs-CRP,High-sensitivity C-reactive protein;EO,Eosinophil;LYMPH,Lymphocyte;MONO,Monocyte;NEUT, Neutrophil;PLT,Platelet;NLR,Neutrophil-to-Lymphocyte Ratio;NPAR,Neutrophil Percentage to Albumin Ratio;FIB,Fibrinogen.

### Relationship between NPAR and pelvic adhesions

Logistic regression analysis was used to evaluate the association between NPAR levels and pelvic adhesions. The results demonstrated that, when analyzed as a continuous variable, NPAR exhibited significant positive associations with moderate-to-severe pelvic adhesions across all models. In the unadjusted model (Model 1), NPAR showed a significant association with adhesion risk (OR = 1.67, 95% CI: 1.40–1.99, *P* < 0.001). This association remained stable in Model 2 (OR = 1.65, 95% CI: 1.38–1.98, *P* < 0.001), strengthened in Model 3 (OR = 1.84, 95% CI: 1.42–2.39, *P* < 0.001), and persisted significantly in the fully adjusted Model 4 (OR = 1.82, 95% CI: 1.39–2.38, *P* < 0.001). Each 10-unit increase in NPAR corresponded to an 82% elevated risk of moderate-to-severe pelvic adhesions. When NPAR was analyzed by tertiles, patients in the highest tertile (T3) had significantly higher risks of moderate-to-severe pelvic adhesions compared to the lowest tertile (T1): Model 1: OR = 4.89 (95% CI: 2.45–9.73, *P* < 0.001), Model 2: OR = 4.49 (95% CI: 2.22–9.08, *P* < 0.001), Model 3: OR = 3.12 (95% CI: 1.21–8.03, *P* = 0.018), Model 4: OR = 2.86 (95% CI: 1.09–7.53, *P* = 0.033). A significant dose-response trend was observed (*P* for trend = 0.042, Table 2). Furthermore, logistic curve-fitting analysis demonstrated a consistent positive linear relationship (non-curvilinear, *P* = 0.095) between NPAR and pelvic adhesion severity, as illustrated in S1 Fig.

**Table 2:** Relationship between NPAR and the Severity of Pelvic Adhesions.

Variable	Model 1		Model 2		Model 3		Model 4	
	OR (95%CI)	*P*-value	OR (95%CI)	*P*-value	OR (95%CI)	*P*-value	OR (95%CI)	*P*-value
**NPAR**	1.67 (1.4 ~ 1.99)	<0.001	1.65 (1.38 ~ 1.98)	<0.001	1.84 (1.42 ~ 2.39)	<0.001	1.82 (1.39 ~ 2.38)	<0.001
NPAR Tertiles*								
T1 (n = 82)	1 (Ref)	1 (Ref)	1 (Ref)	1 (Ref)	1 (Ref)	1(Ref)	1 (Ref)	1 (Ref)
T2 (n = 82)	1.72 (0.93 ~ 3.18)	0.087	1.67 (0.89 ~ 3.13)	0.111	1.28 (0.63 ~ 2.58)	0.492	1.28 (0.63 ~ 2.59)	0.491
T3 (n = 82)	4.89 (2.45 ~ 9.73)	<0.001	4.49 (2.22 ~ 9.08)	<0.001	3.12 (1.21 ~ 8.03)	0.018	2.86 (1.09 ~ 7.53)	0.033
***P* for Trend**		<0.001		<0.001		0.024		0.042

NPAR: Neutrophil Percentage to Albumin Ratio;OR: Odds Ratio;CI: Confidence Interval;Ref: Reference.

Model 1: Unadjusted.

Model 2: Adjusted for age, BMI, gravidity, and parity.

Model 3: Further adjusted for lymphocyte count, neutrophil count, platelet count, and fibrinogen.

Model 4: Further adjusted for CA125 and NLR.

### Sensitivity analysis

**Fig 1:**
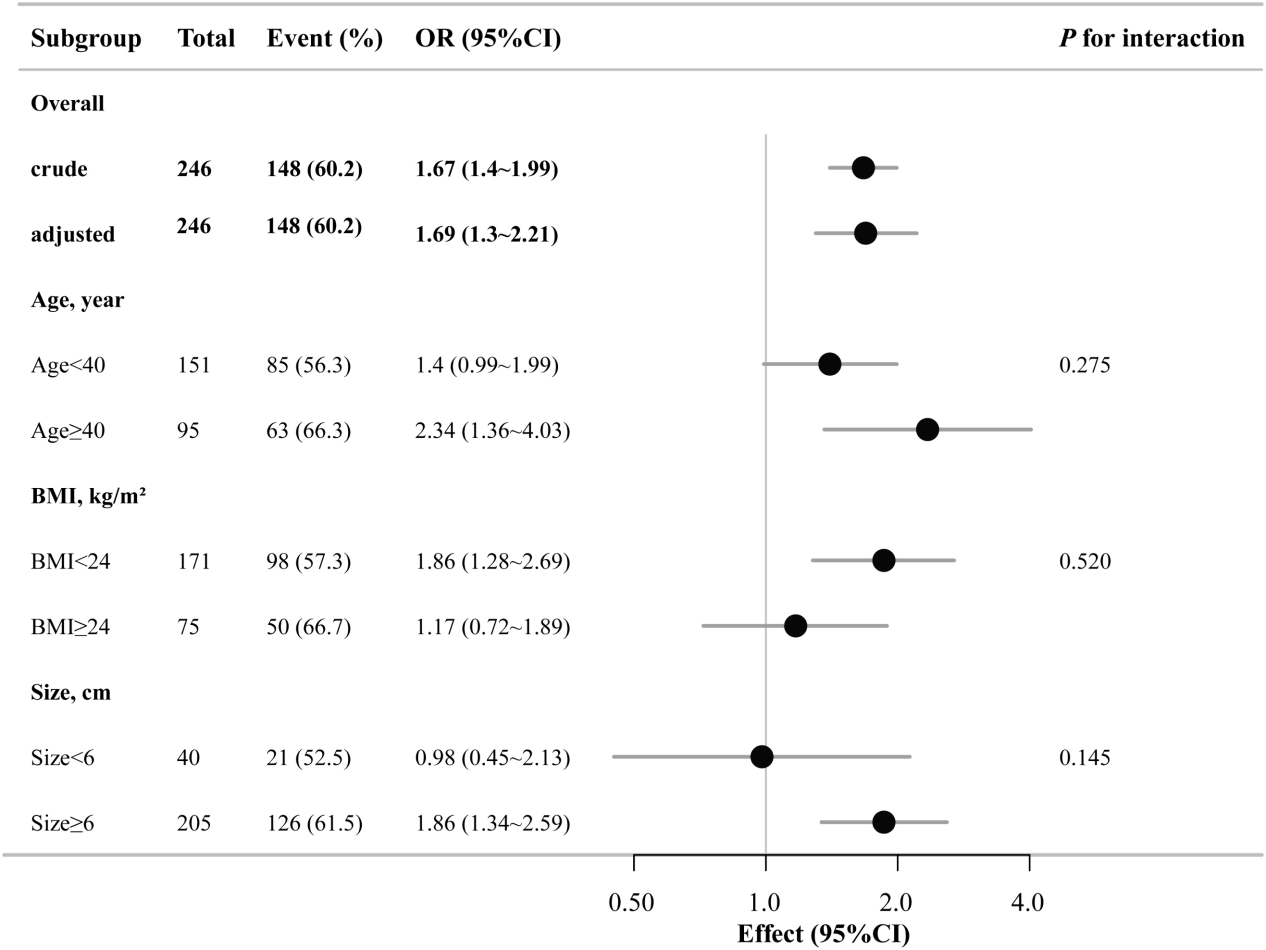
Forest plot of the odds ratios for moderate-to-severe pelvic adhesions in different subgroups (age, BMI, cyst size). Adjusted for age, gravidity, parity, body mass index (BMI), lymphocyte count, neutrophil count, platelet count, fibrinogen, NLR, and CA125. CI: Confidence Interval, OR: Odds Ratio.

Sensitivity analyses were performed using subgroup analyses (stratified by age, BMI, and cyst size) to evaluate the robustness of the association between NPAR and pelvic adhesion severity. Subgroup analyses confirmed that NPAR was a significant predictor of pelvic adhesion severity, and the results remained consistent across strata (Fig 1). No significant interaction effects were observed between NPAR and the stratification factors (all *P* > 0.05, Fig 1), indicating the robustness of the association.

### ROC curve analysis

**Fig 2:**
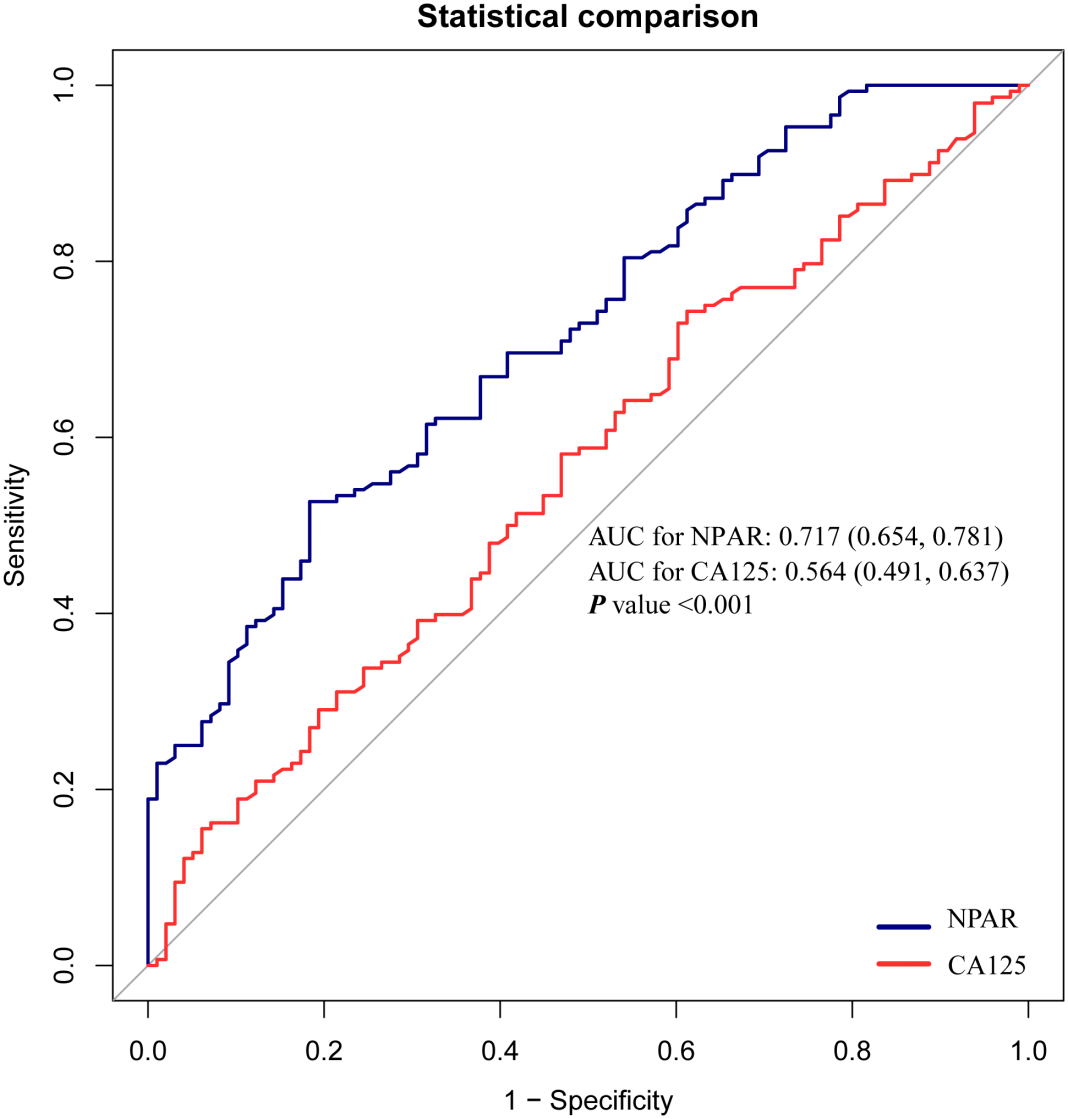
ROC Curve Analysis to Assess the Predictive Performance of NPAR and CA125 for Moderate-to-Severe Pelvic Adhesions in Endometriosis.

ROC curve analysis showed that NPAR (AUC = 0.717) had a significantly better predictive performance for moderate-to-severe adhesions than CA125 (AUC = 0.564; *P* < 0.001) (Fig 2).

## Discussion

Endometriosis is a chronic inflammatory disease. The biological rationale for NPAR as a biomarker stems from the systemic inflammatory response’s combined effects on its two components. On one hand, the chronic inflammatory state characteristic of endometriosis promotes neutrophil activation. These activated neutrophils infiltrate the peritoneal environment and release a cascade of pro-fibrotic factors, including reactive oxygen species, matrix metalloproteinases, and neutrophil extracellular traps [13]. These mediators not only directly cause tissue damage but also perpetuate a pro-fibrotic microenvironment, stimulating fibroblasts to deposit excessive collagen and thereby initiating and advancing adhesion formation [14]. On the other hand, the same systemic inflammatory process may lead to a relative decrease in serum albumin levels. Chronic inflammation may suppress hepatic albumin synthesis while increasing capillary permeability and catabolic metabolism [15]. Although albumin levels typically remain within the clinically normal range, this relative decrease is significant as it may reflect diminished intra-abdominal anti-inflammatory and antioxidant capacity. Albumin normally contributes to maintaining colloid osmotic pressure and scavenging free radicals; thus, its relative reduction may promote tissue edema, impair clearance of fibrin deposits, and exacerbate oxidative stress, collectively facilitating the development of permanent adhesions [16]. Thus, NPAR integrates two pathophysiological pathways: an elevated neutrophil percentage representing active pro-fibrotic signaling, and a relative reduction in albumin indicating compromised anti-inflammatory and tissue repair reserves. Together, these create a peritoneal environment highly susceptible to adhesion formation.

According to the classification of the American Society for Reproductive Medicine (ASRM), endometriosis is divided into four clinical stages based on the size of the ectopic lesion during surgery, the characteristics of its location, severity of the lesion, and the degree of adhesions [17]. There is generally a positive correlation between the ASRM stage and pelvic adhesions, which are present throughout the onset and progression of endometriosis and worsen as the disease progresses. However, the degree of pelvic adhesions and severity of endometriosis symptoms are inconsistent and may even be inversely related. Pelvic adhesions are not only an important cause of infertility in patients with endometriosis but may also lead to postoperative recurrence by obstructing the surgical field and increasing the risk of residual lesions. Advanced disease stage and incomplete first surgery may be high-risk factors for recurrence [18]. While the thoroughness of the first operation depends largely on the severity of adhesions, and inadequate visualization may lead to incomplete removal of endometriotic lesions, resulting in a higher recurrence rate. Thus, adhesions play an important role in endometriosis recurrence. Ideally, it is better for patients with endometriosis to undergo surgery only once in their lifetime. However, there are no standardized criteria or consensus on the timing of surgery. Therefore, preoperative assessment of the degree of adhesion is particularly important. However, there is no well-established test that adequately assesses the severity of pelvic adhesions preoperatively.

Our study found that NPAR was positively associated with the severity of pelvic adhesions, and the logistic proportional risk regression model showed a statistically significant difference for moderate-to-severe pelvic adhesions in all models when NPAR was used as a continuous variable in the fully adjusted model (OR = 1.82, 95% CI: 1.39–2.38, *P* < 0.001). As a categorical variable, the risk of severe pelvic adhesions significantly increased in the highest tertile of NPAR. Compared with the lowest level (T1), there was a trend of an increasing risk of moderate-to-severe pelvic adhesions with increasing NPAR levels, and the test for trend was statistically significant (*P* for trend = 0.042). The logistic fit curve analysis demonstrated a positive linear trend, consistent with the results of the logistic regression model were positively correlated. However, the study by Dominoni et al. did not find a statistically significant difference between the stage of endometriosis and CA125 [19]. Interestingly, the efficacy of NPAR in predicting pelvic adhesions was assessed in this study by ROC curve analysis, which demonstrated that the area under the curve for the NPAR score was 0.717, whereas the area under the curve for the CA125 score was 0.564. This result suggests that CA125, as a classical serological index, has a predictive ability for pelvic adhesions, although it has high sensitivity and specificity in the diagnosis of endometriosis. NPAR is a novel biomarker for assessing the severity of pelvic adhesions. Our study observed a significant incline in the risk of moderate-to-severe pelvic adhesions with increasing levels of NPAR, which may be closely related to the inflammatory response of endometriotic tissue to surrounding tissues and the fibrotic process. Elevated NPAR may promote the fibrotic pathway by activating the neutrophil-mediated inflammatory response, possibly reflecting the progression of the pathologic state, indicating that clinicians can incorporate NPAR as a valuable reference indicator when evaluating disease severity. This suggests that NPAR may be valuable for preoperative assessment of pelvic adhesion severity in patients with endometriosis.

Medication is usually the first choice for patients with mild diseases who do not wish to become pregnant immediately. For late-stage women with fertility needs, surgery does not improve pregnancy rates but may instead damage ovarian function as a result of surgery [20]. Individualized treatment is formulated after adequate preoperative evaluation through NPAR and available imaging, and careful consideration should be provided to offer assisted reproductive technology to accomplish fertility as early as possible, rather than preferring surgical treatment [21]. Furthermore, initial preoperative evaluation via simple and easily accessible blood tests allows for better preoperative preparation, including preoperative bowel preparation, selection of surgical modalities, prevention and management of complications, communication with the patient regarding the condition, and judgment of the postoperative prognosis in patients with fertility requirements.

Our study confirmed for the first time an independent correlation between NPAR and severe pelvic adhesions in patients with endometriosis. Multivariable logistic regression analysis and logistic curve fitting analysis were used to confirm the relationship between NPAR and pelvic adhesions We also performed a sensitivity analysis with robust results, providing a new approach for the noninvasive assessment of disease severity. However, this study has some limitations. First, as a retrospective study, the pelvic adhesion score was based on the subjective recordings of the surgeons, which may be subject to data collection bias. Second, the changes in blood analyses and NPAR involved inflammatory processes; therefore, all other acute inflammatory processes must be excluded before they can be used as a tool for predicting pelvic adhesions in patients with endometriosis, and bias may also occur in the process of exclusion. In addition, the sample size of this study was limited and from a single medical institution. The sample size and single-center nature of the selection may have an impact on the generalizability of the results. Therefore, subsequent studies should expand the sample size and consider prospective studies with more diverse patient populations to confirm the clinical utility of this study.

## Conclusions

In conclusion, our study demonstrates that NPAR is an independent correlate of pelvic adhesions severity in endometriosis patients and exhibited a moderate predictive ability (AUC = 0.717) for moderate-to-severe disease. This indicates its potential as a supplementary tool for preoperative risk stratification, though future prospective, multicenter studies are warranted to verify its generalizability and define its precise role in clinical practice.

## Supporting information

S1 Fig Dose-response Relationship between NPAR and the Severity of Pelvic Adhesions. (PDF)

S1 Data Minimal data set. (XLSX)
