# Supplementary material for: Association between the neutrophil percentage-to-albumin ratio and pelvic adhesion severity in endometriosis: A retrospective cross-sectional study
Source: PLoS One. 2025 Dec 2;20(12):e0337077. doi: 10.1371/journal.pone.0337077 (PMC12671820; doi:10.1371/journal.pone.0337077)
Supplement: S1 Fig — (PDF) [file pone.0337077.s001.pdf]

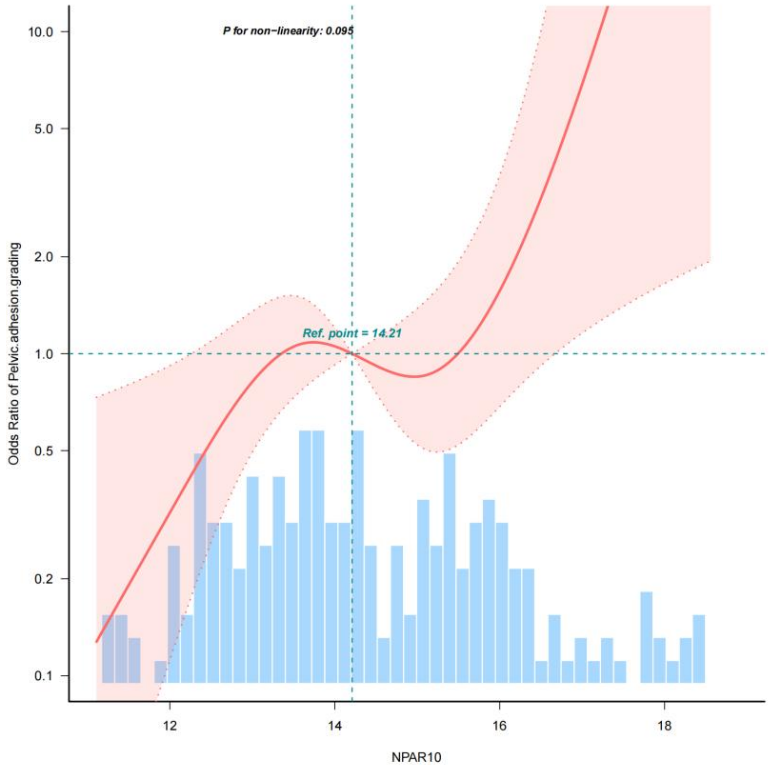

S1 Fig Dose-Response Relationship between NPAR and the Severity of Pelvic Adhesions. Adjusted for age, gravidity, parity, body mass index (BMI), lymphocyte count, neutrophil count, platelet count, fibrinogen, NLR, and CA125. The solid red line and dashed pink lines represent the estimated values and their corresponding 95% confidence intervals, respectively.
